# Modelling mortality within 28 days among preterm infants at a tertiary hospital in Lusaka, Zambia: a retrospective review of hospital-based records

**DOI:** 10.11604/pamj.2021.39.69.27138

**Published:** 2021-05-25

**Authors:** Moses Mukosha, Patrick Kaonga, Kunda Mutesu Kapembwa, Patrick Musonda, Bellington Vwalika, Mwansa Ketty Lubeya, Choolwe Jacobs

**Affiliations:** 1 Department of Pharmacy, University of Zambia, Lusaka, Zambia,; 2 School of Public Health, University of Zambia, Lusaka, Zambia,; 3 Neonatal Intensive Care Unit, Women and Newborn Hospital, Lusaka, Zambia,; 4 Department of Obstetrics and Gynecology, University of Zambia, Lusaka, Zambia,; 5 Young Emerging Scientists Zambia, Lusaka, Zambia

**Keywords:** Neonates, mortality, preterm infants, modelling, Zambia, Neonatal Intensive Care Unit (NICU)

## Abstract

**Introduction:**

globally, almost half of all deaths in children under five years of age occur among neonates. We investigated the predictors of mortality within 28 days among preterm infants at a tertiary hospital in Lusaka, Zambia.

**Methods:**

we reviewed admission records linked to birth, mortality, and hospital discharge from 1^st^ January 2018 to 30^th^ September 2019. Information was retrieved with a follow-up period of 28 days post-delivery to discharge/mortality. We used the Weibull hazards regression to establish the best predictor model for mortality among the neonates.

**Results:**

a total of 3237 case records of women with a median age of 27 years (IQR, 22-33) were included in the study, of which 971 (30%) delivered term infants and 2267 (70%) preterm infants. The overall median survival time of the infants was 98 hours (IQR, 34-360). Preterm birth was not associated with increased hazards of mortality compared to term birth (p=0.078). Being in the Kangaroo Mother Care compared to Neonatal Intensive Care Unit (NICU), and a unit increase in birth weight were independently associated with reduced hazards of mortality. On the other hand, having hypoxic-ischemic encephalopathy, experiencing difficulty in feeding and vaginal delivery compared to caesarean section independently increased the hazards of mortality.

**Conclusion:**

having hypoxic-ischemic encephalopathy, vaginal delivery, and experiencing difficulty in feeding increases the risk of mortality among neonates. Interventions to reduce neonatal mortality should be directed on these factors in this setting.

## Introduction

Preterm birth (PTB) is the birth of a baby before 37 completed weeks of gestation and is a global burden with associated complications which remain high [1, 2]. Globally, approximately 15 million PTB occur each year, and at least 1 million die due to complications of prematurity [3]. The burden of neonatal mortality is highest in low and middle-income countries (LMICs) compared to developed countries, and the trends have been on the increase [4]. Studies have reported that in LMICs, nearly 50% of the babies born at or below 32 weeks die mainly due to inadequate implementation of World Health Organisation (WHO) low cost but highly effective interventions. Some of these interventions include antenatal corticosteroids in women presenting with preterm labour, Kangaroo Mother Care (KMC) and adequate use of antibiotics in preterm babies with a septic risk [5]. In contrast, in high-income countries, nearly all preterm infants with similar age survive [6].

In developing countries, neonatal mortality average around 26 per 1000 live births in contrast, just 3 per 1000 live births in developed countries [7, 8]. For instance, in the United States, the rate is reported around 1.3% per year [9], while a study done in Kenya, reported that nearly 50% of the preterm babies admitted in one of the referral hospitals died [10]. Similarly, Zambia shares the disproportionate burden of PTBs within sub-Saharan Africa (SSA) [11], with about 37.5% of the neonatal mortality due to PTB [12]. Globally, an estimated 2.9 million newborns are expected to die within the first four weeks of life [13] and the reasons could partly be that preterm infants experience a lot of complications such as respiratory distress syndrome (RDS), difficulty in feeding, necrotising enterocolitis (NEC), HIE and sepsis [14-18]. Additionally, HIV exposed preterm infants have been shown to be at increased risk of mortality [19]. Nevertheless, prevention of mother to child transmission of HIV using antiretroviral therapy has reversed this trend in the recent past [20-22].

In Zambia, a number of interventions have been adopted to improve survival of preterm infants such as administration of antenatal corticosteroids like dexamethasone in women presenting with preterm labour, neonatal resuscitation, and KMC which involves the mother and neonate in skin to skin contact and adequate breastfeeding [11]. Additionally, a Low-cost Infection Control Intervention Bundle was recently tested at the study setting whose aim was to reduce the number of infections and subsequent mortality among neonates [23]. However, despite the implementation of these preventive measures, in Zambia, PTB still contributes about 35% of neonatal deaths every year [12, 24]. And yet, the predictors of mortality among PTB are still not well understood [11, 24]. Understanding the predictors of mortality among preterm infants is necessary to inform clinical management and care, provide information to parents, and also target and evaluate interventions. We, therefore, set out to assess predictors of mortality among preterm infants at Women and Newborn Hospital in Lusaka, Zambia, to understand and possibly improve the survival rates of preterm infants.

## Methods

**Study design**: this was a retrospective cohort study. The study used admission records from 1^st^ January 2018 to 30^th^ September 2019 with linked birth, mortality, and hospital discharge records. The interest was to identify preterm infants from admission to determine their survival rates past 28 days from admission to NICU ward. Preterm infants who survived past 28 days or discharged before 28 days were right-censored [25] while all those who experienced the event (mortality) were classified as “failure”.

**Study setting**: the study was conducted in NICU and Kangaroo Mother Care (KMC) admission ward at the Women and Newborn Hospital in Lusaka, Zambia. This is the largest referral hospital in Zambia for both obstetric and gynaecological conditions [26]. On average, 28,800 pregnant women per year are seen, and about 18,000 births/year are recorded at this hospital. The average neonatal admissions per year are above 4000 [23]. The hospital receives referrals from over 20 clinics and five first-level hospitals from surrounding areas of Lusaka and other parts of the country. Asphyxiated babies admitted to the NICU are presumed septic and given antibiotics upon admission to the neonatal unit. Blood cultures are done prior to antibiotics when available for babies presumed septic or have a septic risk.

**Figure 1:**
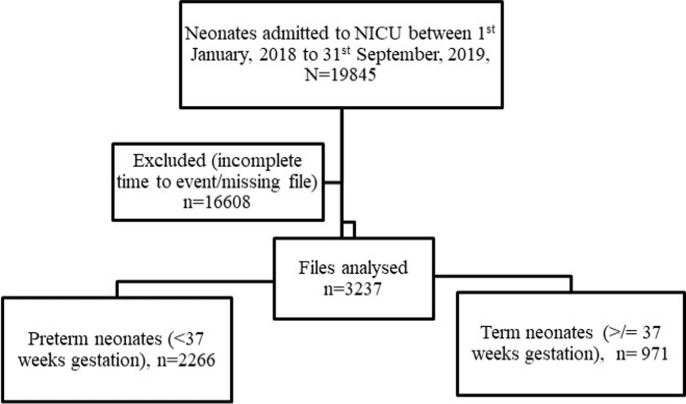
flow chart of the sampling frame for the records

**Study population and sampling procedure**: the study population comprised neonates who were admitted to the NICU and those sent to KMC ward, with complete follow-up time from birth to either discharge or mortality. We defined a neonate as a newborn infant less than 28 days of life [27]. These were further classified as term (< 37 completed weeks of gestation) and preterm infants (< 37 completed weeks of gestation) [28]. We included neonates with complete records (time from admission to time of mortality or discharge from the ward) and a minimum of 90% information for required variables recorded. We excluded neonates born from mothers who delivered in other clinics and hospitals. Overall, the “de facto” eligible sample amounted to 3237 records (Figure 1).

**Sample size and power analysis**: a preliminary study conducted in Zambia reported 37.2% mortality among neonates attributed to preterm births and 60% to other causes, and this corresponded to 1.5% (16/1754) and 9.1% (27/1754) respectively from the total mortality in the study population. Using the freedman method [29] on the assumption of minimum power 80% the number of eligible medical records were sufficient to detect at least 7.6% difference in mortality between the preterm infants and term infants with precision 95% and a significance level of 5%.

**Data extraction**: data was collected from Neonatal Case Record (NCR) and admission files using an Excel-based predesigned data collection form by trained assistants. Completeness of information on all variables as recorded in the files was audited at the end of each day to ensure accuracy. Relevant demographic and medical data were extracted from the medical records. Demographic data included maternal age, sex and age of baby (in hours), on admission. For medical data, we recorded birth weight, gestational age, Apgar score, feeding status, care of preterm infants, diagnosis of sepsis, kangaroo mother care, diagnosis of hypoxic-ischaemic encephalopathy, mode of delivery and HIV exposure status. The extracted data was then entered into Microsoft excel operating system. Data coding, cleaning and validation were conducted until the database corresponded with data collected on the forms. After that data was exported to Stata (version 16) in readiness for analysis

### Study outcome

**Mortality**: interest was to model predictors of neonatal mortality with time to the event (mortality). Information on RDS, sepsis, KMC, feeding status, HIE, mother's HIV status, gestational age, NEC and birth weight were used to control for confounding.

**Statistical methods**: continuous variables such as birth weight of the baby and maternal age were tested for normality using Shapiro-Wilk test and Quantile-Quantile plots therefore median, and interquartile range (IQR) were reported for descriptive statistics because the variables were skewed, while for categorical variables, frequencies and percentages were reported. For the comparison of baseline characteristics, Wilcoxon rank-sum test (Mann-Whitney test) was used for maternal age and birth weight. We used Pearson chi-square test of independence for categorical variables after checking the assumption of a chi-square test (expected cell frequency should not be less than 5). The effect of preterm birth and other predictors on mortality was estimated using adjusted Weibull model. This model was used given that the assumptions of constant hazard overtime for a Cox model was not satisfied. In order to find a Weibull regression model, firstly backward elimination with a liberal P-value for exclusion of P=0.20 was carried out in order to identify clinical and socio-demographic factors to include in the final model. The main primary exposure (preterm birth) was then fixed in the model with the identified clinical and socio-demographic factors. The model was then subjected to a backward elimination selection with a stricter P-value for exclusion of P=0.10. Appropriate model fitting parameters and assumptions were checked after fitting the final model. The Kaplan-Meier survival curve was used to illustrate the difference in survival between preterm and term neonates. Inflation of type-I error rate was avoided, loss of power and classification of individuals who are close to the chosen cut point as having very different levels of risk, by not categorising continuous predictor variables (such as birth weight) [30]. All statistical tests were conducted at the significance level of alpha 0.05 and 95% confidence intervals. Data were analysed using Stata/IC version 16 (Stata Corporation, College Station, Texas, USA).

**Ethical considerations**: the University of Zambia Biomedical Research Ethics Committee UNZABREC approved this study (ref: UNZA-221/2019). Additional permission was obtained from Women and Newborn Hospital management to extract data and conduct the study at the hospital. Data were de-identified to protect the participant's confidentiality.

## Results

**Clinical and demographic characteristics of study participants**: the study reviewed 3237 medical records of neonates with case records of the mother's delivery notes. Majority 1854 (55.7%) of the neonates were males. About 2366 (71.2%) were delivered vaginally, 953 (38.8%) through caesarean section. From the total, 584 (18%) were exposed to HIV infection, 926 (27.9%) developed Hypoxic Ischaemic Encephalopathy (HIE), 1528 (46.5%) had RDS. Furthermore, 1501 (45.1%) developed sepsis, 329 (10%) had difficulty in feeding, 1162 (35%) were in KMC and 167 (5%) developed NEC. The median maternal age was 27 years (IQR, 22-33). From a total of 3237, 1963 died for whom there was information on time in hours until the event of death. Assuming hazard function to be constant, the incident rate of mortality from a total person-time follow up of 392340 hours was estimated as 0.0049 per hour or 4.9 per 1000 person-hours, which would correspond to 0.0048976*24=0.12 per day or 120 per 1000 persons per day. The median survival time of the neonates was 98 hours (IQR, 34-360). There was a significant difference in birth weight in kilograms (2.4 vs 1.7, p<0.0001) between the infants who did not die and those who died respectively. Additionally, there was evidence of a difference in mortality and mode of delivery (p<0.0001), HIE (p<0.0001), KMC (p<0.0001), NEC (p<0.0001), Gestational age (p<0.0001). On the other hand, exposure to HIV infection (p=0.795), RDS (p=0.484) sepsis (p=0.836), difficult feeding (p=0.316) were not significantly associated with mortality (Table 1).

**Table 1:** clinical and demographic characteristics of neonates by mortality status

Characteristic	Level	Alive n=1274	Died n= 1963	P-value
**Gestational age**	≥37 weeks	492(50.7)	479(49.3)	< 0.0001b
	< 37 weeks	782(34.5)	1484(65.5)	
**Age of mother(years)**	Unit increase	26(22-32)	26(21-33)	0.2053a
**Sex**	Male	741(41.4)	1050(58.6)	0.009b
	Female	533(36.9)	912(63.1)	
**Birth weight(kg)**	Unit increase	2.4(1.6-3.2)	1.7(1.2-2.8)	<0.00001a
**Mode of delivery**	Caesarean section	450(47.8)	491(52.2)	
	Vaginal	819(35.8)	1471(64.2)	<0.0001b
**Exposure to HIV**	No	1017(39.2)	1576(60.8)	
	Yes	221(38.6)	351(61.4)	0.795b
**Hypoxic Ischaemic Encephalopathy**	No	974(42)	1347(58)	
	Yes	298(32.8)	611(67.2)	<0.0001b
**Respiratory Distress Syndrome**	No	695(40)	1046(60.1)	
	Yes	578(38.7)	915(61.3)	0.484b
**Diagnosis of sepsis**	No	718(54.6)	1107(55)	
	Yes	597(45.4)	907(45)	0.836b
**Difficulty in feeding**	No	118(36.8)	203(63.2)	
	Yes	1156(39.6)	1760 (60.4)	0.316b
**Kangaroo Mother Care**	No	731(34.6)	1385(65.5)	
	Yes	543(48.5)	577(51.6)	< 0.0001b
**Necrotising enterocolitis**	No	1238(40.3)	1833(59.7)	
	Yes	36(22.2)	126(77.8)	< 0.0001b

Key: IQR-Interquartile range, awilcoxon ranksum test (Mann-Whitney test), bPearson chi square test, values are percentages and frequencies

**Figure 2:**
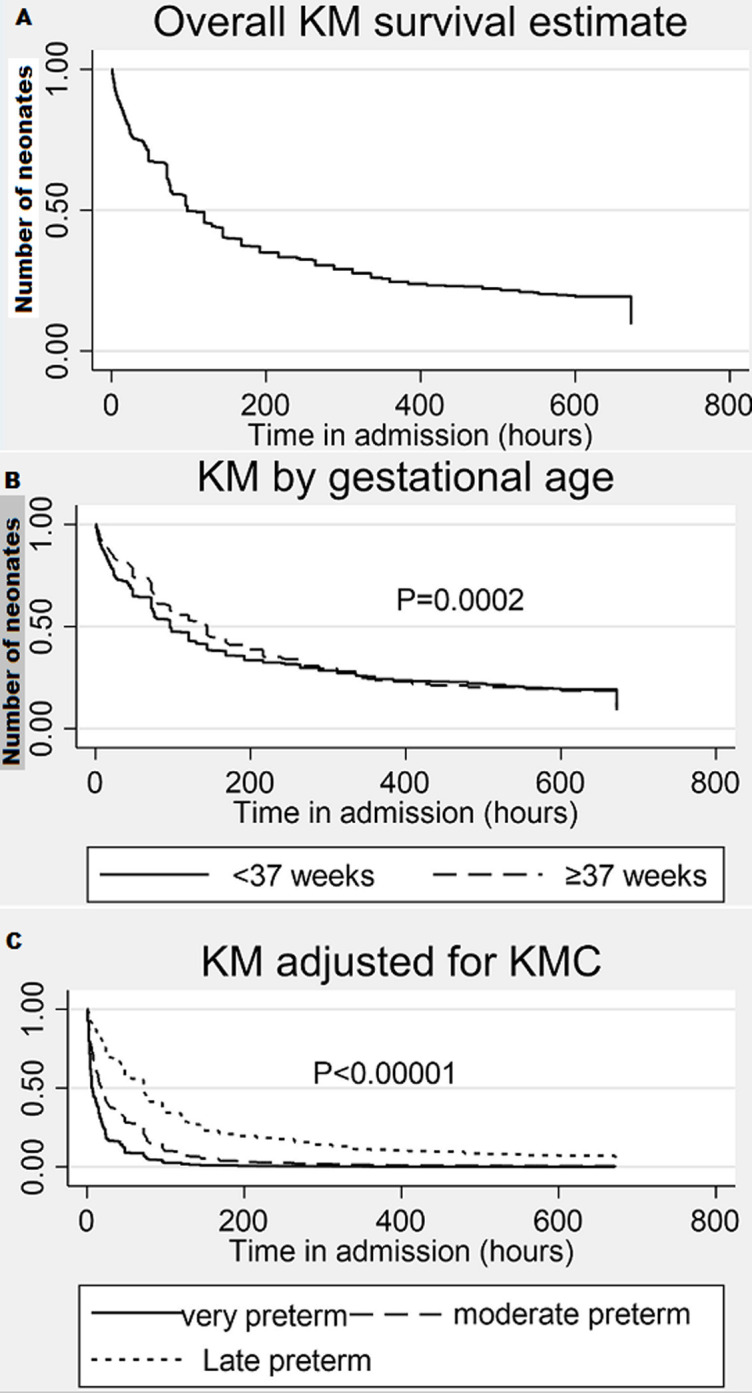
Kaplan-Meier survival curves for neonates admitted to NICU and KMC

The overall Kaplan Meier survival estimate (Figure 2 A) shows that by 600 hours, there were only 143 neonates who were still in the study. Overall, just under 25% of the neonates were alive by 600 hours. We further compared the Kaplan-Meier survival estimate for time to mortality by gestational age category. From the graph (Figure 2 B), term infants appear more likely to survive past 28 days of life compared to preterm infants. To determine the extent of any evidence that term infants survive longer than preterm infants, we additionally tested for the equality of the survival functions in the two groups using the log-rank test albeit and proportional hazard assumption was not satisfied given that the graphs seem to cross each other. Term infants were significantly (p=0.0002) likely to survive longer than preterm infants. On stratified analysis very preterm (less 28 weeks), moderate preterm (28 to 32 weeks) and late preterm (32 to 37 weeks) showed significant differences in survival functions (p<0.00001) when adjusted for KMC (Figure 2C).

**Modelling mortality**: to model mortality, we defined the event of interest as death within 28 days of life from admission into NICU. Any unobserved event was right censored (i.e. discharge, or survival past 28 days). In the following section, we compare two competing models (the Cox-proportional hazards and the Weibull model). The Cox-proportional hazards model is based on the assumption that the hazards (relative risk of mortality) are constant over time (i.e., over the follow-up time of 28 days). The Weibull model assumes that the hazards are not constant but can increase or decrease monotonically [31].

**Figure 3:**
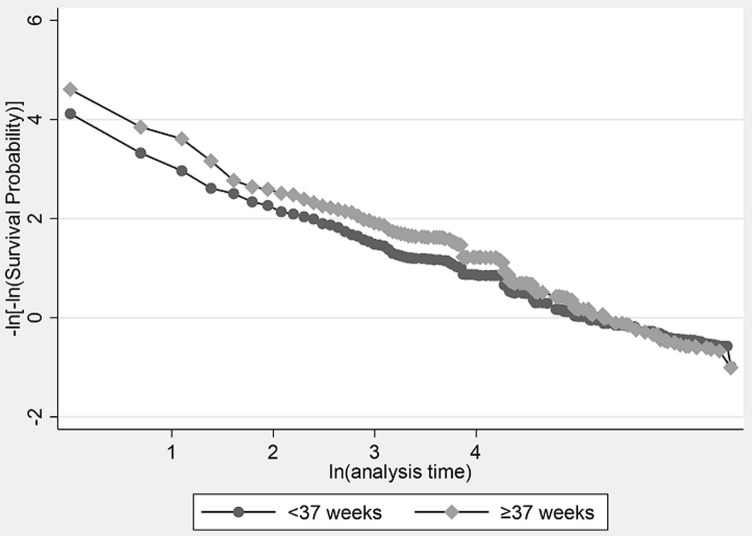
checking the proportional hazards assumption of the Cox proportional regression model

**The cox-proportional hazards model**: the results of the Cox-proportional hazards model are presented (Table 2). The log-likelihood ratio chi-square test LRX^2^_1_ = 312, p<0.00001 for this model indicates that the full model shows a better fit than the null model with no independent variables in predicting the hazards of mortality. However, when tested for the proportional hazards assumption, these were violated in this model because there is an interaction between the lines (Figure 3). Furthermore, the Bayesian Information Criterion (BIC) 27987.12 and Akaike Information Criterion (AIC) 27944.62 were higher than that of the Weibull model (BIC=9359.56, AIC=9304.90). Therefore, the Cox-proportional hazards model is not adequate, and the Weibull model explains the data better in this case (Table 3).

**Table 2:** adjusted predictors of mortality in the first 28 days of life from the Cox-proportional hazards model

Characteristic	Level	AHR (95% CI)	P-value
**Gestational ag**	≥ 37 weeks	1	
	< 37 weeks	1.13(0.99, 1.30)	0.078
**Birth weight (kilograms)**		0.85 (0.77, 0.85)	<0.0001
**Mode of delivery**	Caesarean section	1	
	Vaginal	1.17(1.05, 1.30)	0.003
**Hypoxic Ischaemic Encephalopathy**	No	1	
	Yes	1.53(1.38, 1.69)	<0.0001
**Diagnosis of sepsis**	No	1	
	Yes	0.78 (0.71, 0.85)	<0.0001
**Difficulty in feeding**	No	1	
	Yes	1.18.84 (1.00, 1.39)	0.027
**Kangaroo Mother Care**	No	1	
	Yes	0.61(0.55, 0.67)	<0.000
Goodness-of-fit test overall model (likelihood ratio) Chi-square=312, df=7, p-value <0.00001, BIC=27987.12, AIC=27944.62			

Key: 95% CI=95% confidence interval, AHR= Adjusted Hazard Ratio, BIC=Bayesian Information Criterion, AIC=Akaike's Information Criterion

**Table 3:** adjusted predictors of mortality in the first 28 days of life in from the best model that fits the data well (Weibull model)

Characteristic	level	AHR	P-value	95% confidence interval	
**Gestational age**	≥ 37 weeks (term infant)	1			
	< 37 weeks (preterm infant)	1.12	0.098	0.98	1.29
**Kangaroo Mother Care**	No	1		1	
	Yes	0.59	<0.0001	0.54	0.65
**Difficulty in feeding**	No	1		1	
	Yes	1.19	0.013	1.00	1.40
**Diagnosis of sepsis**	No	1		1	
	Yes	0.77	<0.0001	0.71	0.84
**Hypoxic Ischaemic Encephalopathy**	No	1		1	
	Yes	1.57	<0.0001	1.42	1.74
**Mode of delivery**	Caesarean section	1		1	
	Vaginal	1.18	0.002	1.07	1.31
**Birth weight (kilograms)**	Per unit increase	0.82	<0.0001	0.78	0.86
ln_p*		-0.31	<0.0001	-0.3421979	0.2723112
p*		0.74		0.7102076	0.7616172

Goodness-of-fit test overall model (Likelihood ratio) Chi-square=336, df=7, p-value <0.00001, BIC=9359.56, AIC=9304.90

Key:P*= Ancillary shape parameter of the Weibull model, AHR= Adjusted Hazard Ratio, BIC=Bayesian Information Criterion, AIC=Akaike's Information Criterion, df=degrees of freedom

**Figure 4:**
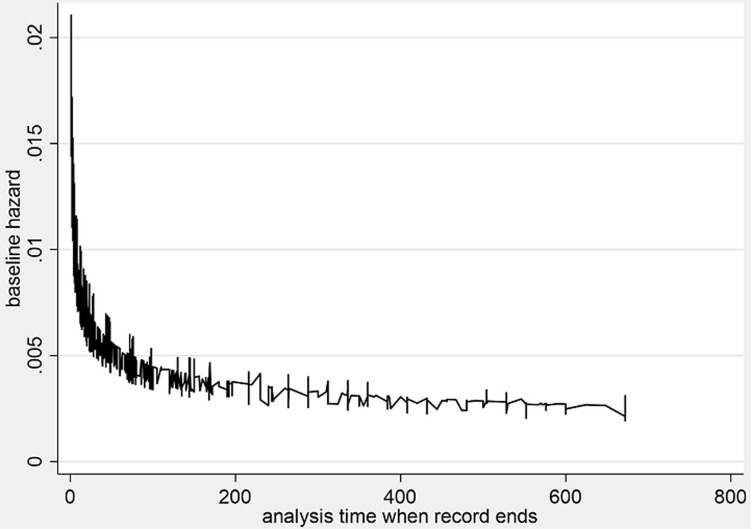
estimated baseline hazard of mortality from the Weibull model

**The weibull model baseline hazard**: the estimated baseline hazard when all the covariates are equal to zero, is shown in Figure 4. We can deduce that as follow-up time increases the hazards of mortality reduces. The goodness of fit test in Table 3 below shows the log-likelihood ratio chi-square test LRX^2^_1_ = 336, p<0.00001 for this model and indicates that the full model shows a better fit than the null model with no independent variables in predicting the hazards of mortality. It further shows a Wald test for H_0_)=: In(p)=0 for which the test statistics is-0.31 and reports that we rejected (p-value<0.0001) the null hypothesis and state that the hazard is not constant. The value parameter P<1 (0.74) indicates that the baseline hazard is monotone decreasing. Therefore, the Weibull model is suitable for modelling this data that is exhibiting monotone hazard rate. From the results of the Weibull model, Being in KMC (AHR; 0.59, 95% CI; 0.54-0.65), having sepsis (AHR: 0.77, 95% CI; 0.71, 0.84), and a unit increase in birth weight (AHR: 0.82, 95% CI; 0.78-0.86) were significantly associated with reduced hazards of mortality controlling for all covariates. On the other hand, having HIE (AHR: 1.57, 95% CI; 1.42-1.74), experiencing difficulty in feeding (AHR: 1.19, 95% CI; 1.00-1.40) and vaginal delivery compared to caesarean section (AHR: 1.18, 95% CI; 1.07-1.31) independently increased the hazards of mortality in this population.

## Discussion

This study was aimed at assessing predictors of mortality among preterm infants at Women and Newborn Hospital in Lusaka, Zambia. The overall median survival of neonates was 98 hours (IQR;34-360). We found that preterm infants were at increased risk of mortality compared to term infants, although we could not rule out random chance finding. Additionally, we found evidence of a difference in risk of mortality between preterm and term neonates and mode of delivery, HIE, KMC, NEC and gestational age. From the Weibull model, we noted that the best predictors of mortality were KMC, difficulty in feeding, sepsis, birth weight, HIE and vaginal delivery. Our results are similar to current evidence on neonatal mortality [32, 33] though we did not find any evidence of the link to exposure to HIV infection and RDS. Consistent with existing literature [2, 7, 34, 35], it was not surprising to find that preterm infants were at risk of mortality in our study setting. One of the reasons could partly be that Zambia still has high levels of PTB, and its subsequent complications could explain the observed hazards of mortality [12]. Most of the complications of preterm birth arise from undeveloped organ systems that are not yet ready to support life outside the uterus environment [36]. The risk of acute neonatal illness and mortality decreases with gestational age, reflecting the fragility and immaturity of the immune system, brain, skin, lungs, kidneys, eyes, and gastrointestinal system [37]. Preterm neonates are prone to various complications such as infections, difficulty in feeding, NEC, RDS [38, 39].

The response of the neonates organ systems to the demands of the extrauterine environment, including NICU have an important impact on the neonates' risk of mortality [35, 36]. According to PK Ndombo, QM Ekei, JN Tochie, MN Temgoua, FTE Angong, FN Ntock and L Mbuagbaw [40], preterm birth complications accounted for the majority (69%) of neonatal mortality in one of the urban hospitals in Cameroon. A five-minute Apgar score less than seven was the only predictor of neonatal mortality in the Cameroonian study. A study in Kenya showed neonatal mortality among preterm infants of 116/200 (58%) in a 7-year period [41] with birth asphyxia being the main predictor of mortality. In Ethiopia 1109/3852 (29%) of preterm infants admitted in NICU between 2016 and 2019 died by 28 days of post-natal age [2]. The main predictors of mortality in the Ethiopian study were RDS, sepsis, hypothermia, asphyxia and pneumonia. The high neonatal mortality rates in Zambia and the sub-Saharan region could make it difficult to attain the SDG target of reducing neonatal mortality to at least as low as 12 deaths per 1000 live births by 2030.

We found a significant difference in birth weight between infants who died and those who did not. Birth weight independently decreased the risk of mortality in the first 28 days of post-natal life. Low-birth-weight of neonates has been linked to early infant mortality previously [42-44]. The WHO recently recommended KMC to be standard of care for preterm infants after evidence showed that it reduced mortality rates among low birth weight neonates [45]. In keeping with WHO report, our study found that neonates who were in KMC had reduced hazards of mortality compared to their counterparts who were not in KMC. KMC has been reported to be an effective way to reduce neonatal mortality due to continuous skin-to-skin contact between mother and baby which improves baby´s temperature, support exclusive breastfeeding and helps early recognition/response to illness [46]. We further found that the infants who were delivered vaginally were more likely to die compared to the ones delivered via CS. Mixed results have been reported in the extant literature. A prospective study by Bishop *et al*. [47] found a higher mortality rate in the neonates delivered through CS. Similarly, Batieha *et al*. [48] found that the rate of CS in Jordan was high (29.1%) and was significantly associated with the risk of neonatal mortality. The biological plausibility of these observations is that if CS is done for severe fetal distress, one is likely to deliver a baby with a low APGAR score and subsequent poor neonatal outcomes. Nevertheless, other studies have reported an inverse correlation between number of CS and neonatal mortality in a given period [49]. This could possibly be explained by several other factors like planned therapeutic CS, which would prevent possible infections associated with vaginal delivery, such as congenital syphilis [19, 50]. Another reason could be that with CS, babies are delivered quickly before they are distressed. This could partly explain the observed differences across different settings.

The findings on HIE in our study support the current evidence in the medical literature that it is a very serious neonatal condition responsible for increased neonatal hospitalisation and mortality [51-54]. This is in line with a prospective study done in Tanzania on HIE proportions, neurological signs and early outcomes of newborns that developed birth asphyxia were they found a mortality rate of 84.2% among the neonates who had severe HIE [55]. HIE is not a single “event” but is rather a cumulative number of processes [56]. Some of the key processes involved in HIE are oxidative stress, inflammatory process, neurotransmitter receptors and excitotoxicity [57]. The clinical signs of HIE suggest a number of molecular events and one plausible remarkable protector of HIE is mild hypothermia which has a neuroprotective effect against ischemic brain injury [58]. Mild hypothermia cools down the cortex resulting in no cerebral haemorrhages and inhibition of harmful cellular processes induced by hypoxic ischaemia [59]. Our study further found a higher likelihood of mortality among neonates who experienced difficulty in feeding. Preterm babies may have feeding intolerance due to immaturity of the gut, and this may result in holding feeds which would result in negative metabolic energy and death if not well handled. Therefore, our findings are within biological plausibility with regards to feeding status. These findings were corroborated by Bjornvad *et al*. [60] who observed that preterm birth and feeding predispose neonates to small intestinal dysfunction, which may lead to NEC and death.

Surprisingly, we found that sepsis was associated with reduced hazards of mortality among neonates in our study. However, it is well documented in the extant literature that sepsis is responsible for increased mortality among neonates in the first 28 days [61-64]. According to the United Nations Children's Fund (UNICEF), among 2.5 million annual neonatal deaths, approximately one-third are due to infections, including sepsis and pneumonia [65]. A multicentre study in India showed a 29% mortality rate among neonates with bloodstream infections compared to 10% among those without the infection [66]. In Cameroonian public hospital, mortality associated neonatal infection was reported at 6% [40]. The above cited studies used prospective study designs and were able to control the accuracy of case definitions which was lacking for our study. Since we used secondary data, the definition of sepsis may not have been accurate, and it could have led to misclassification in our model. For the best predictor model of mortality in NICU at Women and Newborn Hospital, the study found that the Weibull model was most suitable as the baseline hazards of mortality were not constant over the follow-up period but decreased monotonically. Furthermore, the results of the overall model fitting using AIC and BIC favoured the Weibull model. The Weibull models exhibit a wide range of shapes for the density and hazard functions, which makes them suitable for modelling complex failure data sets [67] such as the one we used in our study. We compared the cox proportional hazards model and the Weibull model. The conclusion from the two models was similar in most respect with regards the significant overall predictors and the effect size. The only difference we noted was the parameters of model fitting that showed that the Weibull model was explaining the data well.

This study has limitations worth noting. Firstly, we were unable to verify the consistency of case definitions used for the diagnosis of major conditions known to be associated with neonatal mortality, which may have affected the accuracy of the data recorded. For instance, in the study setting due to shortages of blood culture bottles, the diagnosis of sepsis is mainly presumed and may depend on the level of competence of the attending medical personnel. This may have led to misclassification of neonates in our model and may have had affected the overall results on sepsis. Secondly, we could not control for some maternal variables like prophylaxis with steroids during pregnancy, type of pregnancies (multiple or singleton gestation), diseases during pregnancy, place of delivery, prolonged rupture of membranes, maternal fever, meconium-stained amniotic fluid and premature rupture of membranes as these were not recorded in the files. However, our study has some strength. First, the study used a large data set, giving us adequate power to detect differences in mortality between preterm and term neonates compared to similar studies. Second, we conducted this study in a setting with a very high rate of preterm births and associated complications. This enabled us to assess adequately for various known risk factors of mortality. Additionally, most previous studies have not justified the use of a Cox proportional regression model which is strict on assumptions of constant hazards of mortality over time. Our study was able to demonstrate that the hazards of mortality may actually not be constant over time and regression modelling that takes this aspect into account, like the Weibull model may explain the data well.

## Conclusion

From our findings, the factors associated with risk of mortality among neonates were difficulty in feeding, HIE and vaginal delivery. These findings are suggestive of the need for NICU staff to closely monitor neonates presenting with HIE and manage them promptly to reduce their risk of mortality. Mortality in these neonates is best modelled in the Weibull model that takes into account varying baseline hazards over a follow-up period.

### What is known about this topic

- The neonatal mortality rate is quite high in sub-Saharan Africa;
- The risk of mortality is higher for preterm infants than the term infants;
- Sub-Saharan Africa shares this burden disproportionately when compared to other developed countries.

### What this study adds

- The neonatal mortality rate at a tertiary public health institution in Zambia which is the largest referral hospital for obstetric and neonatology conditions in the country;
- Kangaroo mother care reduces the hazards of mortality within 28 days in the study population;
- The best model that explains neonatal mortality data well is the Weibull regression model.
